# Cysteine 467 of the ASCT2 Amino Acid Transporter Is a Molecular Determinant of the Antiport Mechanism

**DOI:** 10.3390/ijms23031127

**Published:** 2022-01-20

**Authors:** Mariafrancesca Scalise, Gilda Pappacoda, Tiziano Mazza, Lara Console, Lorena Pochini, Cesare Indiveri

**Affiliations:** 1 Unit of Biochemistry and Molecular Biotechnology, Department DiBEST (Biologia, Ecologia, Scienze Della Terra), University of Calabria, Via P. Bucci 4C, 87036 Arcavacata of Rende, Italy; mariafrancesca.scalise@unical.it (M.S.); gilda.pappacoda@unical.it (G.P.); tiziano.mazza@unical.it (T.M.); lara.console@unical.it (L.C.); lorena.pochini@unical.it (L.P.); 2 CNR Institute of Biomembranes, Bioenergetics and Molecular Biotechnologies (IBIOM), Via Amendola 122/O, 70126 Bari, Italy

**Keywords:** amino acid, glutamine, transport, over-expression, liposome, site-directed mutant, 3D structures

## Abstract

The plasma membrane transporter ASCT2 is a well-known Na^+^-dependent obligatory antiporter of neutral amino acids. The crucial role of the residue C467 in the recognition and binding of the ASCT2 substrate glutamine, has been highlighted by structure/function relationship studies. The reconstitution in proteoliposomes of the human ASCT2 produced in *P. pastoris* is here employed to unveil another role of the C467 residue in the transport reaction. Indeed, the site-directed mutant C467A displayed a novel property of the transporter, i.e., the ability of mediating a low but measurable unidirectional transport of [^3^H]-glutamine. This reaction conforms to the main features of the ASCT2-mediated transport, namely the Na^+^-dependence, the pH dependence, the stimulation by cholesterol included in the proteoliposome membrane, and the specific inhibition by other common substrates of the reconstituted human ASCT2. Interestingly, the WT protein cannot catalyze the unidirectional transport of [^3^H]-glutamine, demonstrating an unspecific phenomenon. This difference is in favor of a structural conformational change between a WT and C467A mutant that triggers the appearance of the unidirectional flux; this feature has been investigated by comparing the available 3D structures in two different conformations, and two homology models built on the basis of hEAAT1 and GLT_Ph_.

## 1. Introduction

ASCT2 (SLC1A5) is a membrane transporter of paramount importance in both physiological and pathological conditions due to its involvement in maintaining the homeostasis of some neutral amino acids. Indeed, one of the ASCT2 preferred substrates, i.e., glutamine is required for protein synthesis, energy production, and signaling purposes [1,2,3]. ASCT2 belongs to the SLC1 family that is constituted by two subgroups of proteins: the first one includes five high affinity glutamate transporters (EAATs); the second one includes two neutral amino acid transporters, ASCT1 and ASCT2. Historically, the two subgroups have been described and studied separately because they are distinguished in terms of transport modes, substrate specificities, and tissue distributions [4,5]. EAATs catalyze a unidirectional flux of anionic amino acids, whereas ASCTs catalyze an antiport of mostly neutral amino acids [6,7]. Each subgroup is further characterized by different ion coupling, in terms of both ion type and flux direction: EAATs (SLC1A1, A2, A3, A6, A7) are involved in glutamate and aspartate uptake, coupled to exchange of 3Na^+^_ex_:1H^+^_ex_, with K^+^_in_ [6]. Recently, the involvement of extracellular calcium in the transport process has also been proposed [8]. ASCTs are responsible for the antiport of neutral amino acids, coupled to uptake of Na^+^ [7]. ASCT1 transports alanine, serine, and cysteine, but not glutamine [9,10]; ASCT2 transports glutamine, alanine, and serine, but not cysteine [11,12,13]. The information on ASCT2 derives from studies on intact cells, oocytes expressing the transporter, and proteoliposomes harboring the human protein obtained by over-expression in *P. pastoris* [12,13,14,15]. The reconstitution in proteoliposomes allowed the revealing of some features of this transporter, such as the stimulation by interaction with cholesterol [16], and the functional and kinetic asymmetry for the substrates [17]. Furthermore, the use of the single-protein proteoliposome approach shed new light on some controversial aspects of the ASCT2 function. The transport stoichiometry was established as 2Na^+^_ex_:1aa^0^_ex_:1aa^0^_in_ [18]; this finding, obtained by combining radiometric and fluorometric assays, corroborated the previous observations on the electrogenic nature of the ASCT2 transport reaction, which is slightly stimulated by an imposed membrane potential across membrane [17]. Interestingly, the low extent of stimulation by membrane potential is shared with the hEAAT3 [19] and with the bacterial homologue Glt_Ph_ [20]. Over the years, the molecular determinants of the substrate binding site layout have been described for EAATs and ASCT2 by combining bioinformatics and site-directed mutagenesis. More recently, the resolution of 3D structures of both prokaryotic and eukaryotic amino acid transporters led to a huge amount of novel information on the molecular mechanisms responsible for translocation of substrates [6,21,22,23,24]. In this frame, the 3D structures of EAAT1 and ASCT2 were solved [6,25,26] and a crucial residue has been identified; in particular, an arginine residue for EAATs [25] or a cysteine residue for ASCT2 [27]. So far, the role attributed to these two residues related, mainly, to substrate specificity and recognition, as shown by the lowered affinities towards glutamine of the C467A mutant in the case of ASCT2 [27]. It is worth noting that, very recently, the sharp difference in the specificity between EAATs and ASCT2 has been smoothened by the finding that ASCT2 can also mediate transport of glutamate with a mechanism resembling those of both EAATs and ASCT2 [28]; in line with the ASCT2 transport mode, the transport of glutamate occurs as a Na^+^ dependent antiport with internal glutamine; at the same time, the glutamate uptake is coupled to H^+^ resembling the EAATs transport mode [28]. The specific presence of the C467 residue in ASCT2, and the activity retention of the C467A mutant, led us to hypothesize that this residue may be involved in an additional role, other than the substrate binding. In this study, we provide evidence that a unidirectional transport appears in the C467A mutant, which is absent in the WT isoform, proposing a role for this residue in the antiport mechanism of the hASCT2 transport cycle.

## 2. Results

### 2.1. Reconstitution of C467A Mutant in Proteoliposomes

**Figure 1:**
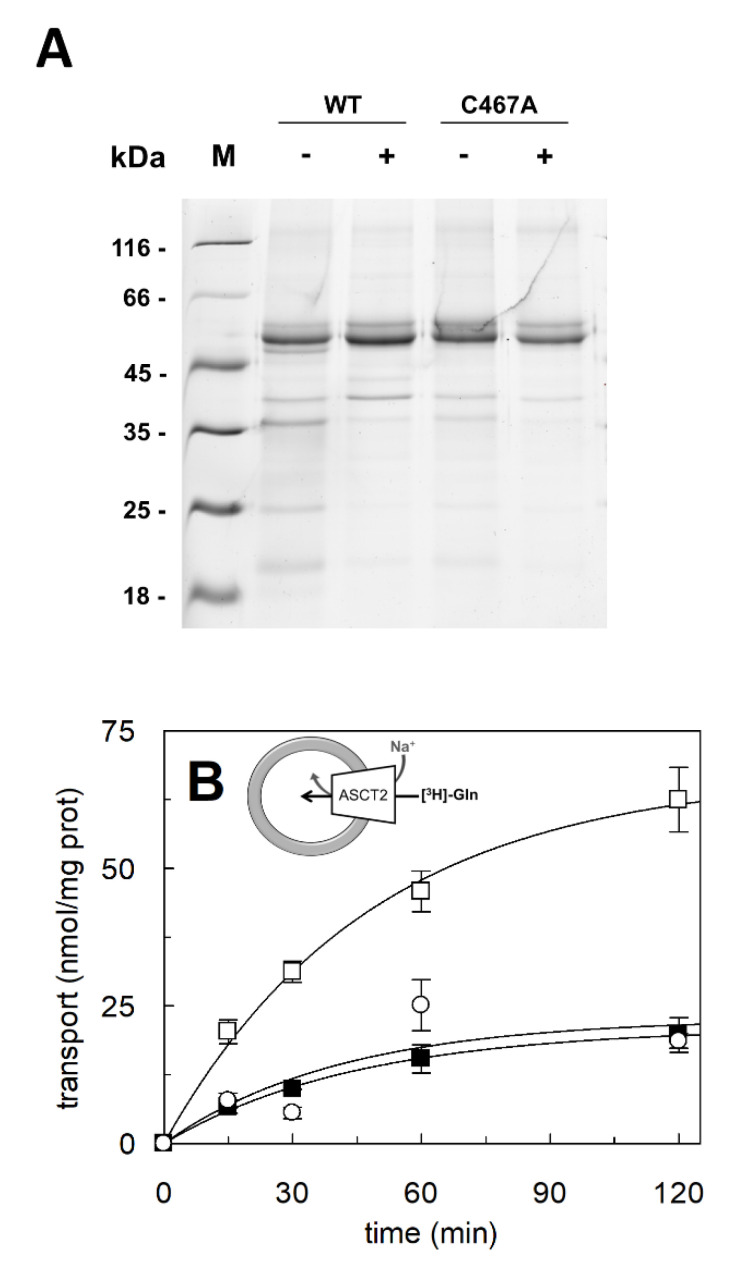

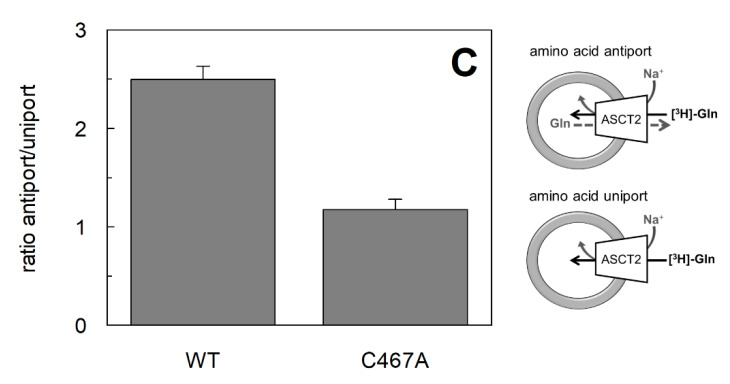
Purification and transport of [^3^H]-glutamine mediated by ASCT2 WT and C467A mutant in proteoliposomes. In (**A**), Blue-Coomassie staining on SDS-PAGE analysis of recombinant hASCT2 WT and C467A purified in the absence or in the presence of 1 mM glutamine in the desalting buffer, as indicated by “+” and “−”. Image is representative of three independent protein preparations. In (**B**), time course analysis of [^3^H]-glutamine unidirectional transport. The proteoliposomes were reconstituted with WT (‛) or C467A (□, ■). The transport was started by adding 500 µM [^3^H]-glutamine to proteoliposomes prepared without internal glutamine, in the absence (■) or in the presence (‛, □) of 50 mM Na-gluconate. The transport was stopped at indicated times according to the stop inhibitor method. In the figure, sketch of the unidirectional transport. In (**C**), ratio of antiport/uniport reactions mediated by WT and C467A reconstituted in proteoliposomes. The transport was started by adding 500 µM [^3^H]-glutamine and 50 mM Na-gluconate to proteoliposomes prepared without internal glutamine (for the amino acid uniport measurements) or 500 µM internal glutamine (for the amino acid antiport measurements). The transport was stopped after 120 min, i.e., at equilibrium. In the figure, sketch of the amino acid antiport or uniport reaction. In (**B**,**C**), results are means ± S.D. from three independent experiments.

To investigate the role of the C467 residue in the antiport reaction mediated by ASCT2, a reliable comparison between the antiport and the unidirectional transport activities was mandatory. To address this point, the mutant C467A and the WT proteins were purified using a glutamine free elution buffer, as previously reported [15]. The glutamine-free protein preparation was necessary to form proteoliposomes with no glutamine in the internal compartment. The amount of WT and C467A proteins, purified using the glutamine-free elution buffer, was comparable with that of the canonical preparations obtained with the glutamine containing buffer [16] (Figure 1A). Then, the glutamine free WT and C467A proteins were reconstituted in proteoliposomes and transport activity was measured as uptake of [^3^H]-glutamine in the absence or in the presence of internally added glutamine (Figure 1B,C). As shown in the time course analysis of Figure 1B, the C467A mutant can mediate a unidirectional transport of [^3^H]-glutamine, with a transport rate of 1.4 ± 0.16 nmol⋅min^−1^⋅mg protein^−1^ much higher than that measured for the WT of 0.4 ± 0.03 nmol⋅min^−1^⋅mg protein^−1^. To better evaluate the features of the C467A unidirectional transport reaction, the [^3^H]-glutamine uptake was measured in the absence of external Na^+^. In this condition, the unidirectional transport mediated by C467A overlaps with that which was mediated by the WT in the presence of external Na^+^; this suggests that the unidirectional glutamine flux observed in the case of WT may be an unspecific phenomenon rather than a canonical transporter-mediated process. Finally, to compare the antiport transport and the unidirectional transport catalyzed by WT and C467A, the ratio antiport/uniport was assessed (Figure 1C). To obtain a reliable comparison of the two transport modes, the conventional in/out gradient of glutamine concentration, established to generate accumulation of [^3^H]-glutamine inside [15], was abolished. To achieve this objective, the external and internal glutamine concentrations were kept equal in the antiport assay. In good agreement with previously reported data [27], the antiport reaction mediated by WT or C467A was comparable. However, the ratio antiport/uniport catalyzed by the two proteins was substantially different, being 1.2 ± 0.1 for C467A, 2.5 ± 0.13 for the WT, confirming that the antiport and the unidirectional transport almost overlapped in the case of C467A. 

### 2.2. Functional Characterization of C467A Unidirectional Transport in Proteoliposomes

**Figure 2:**
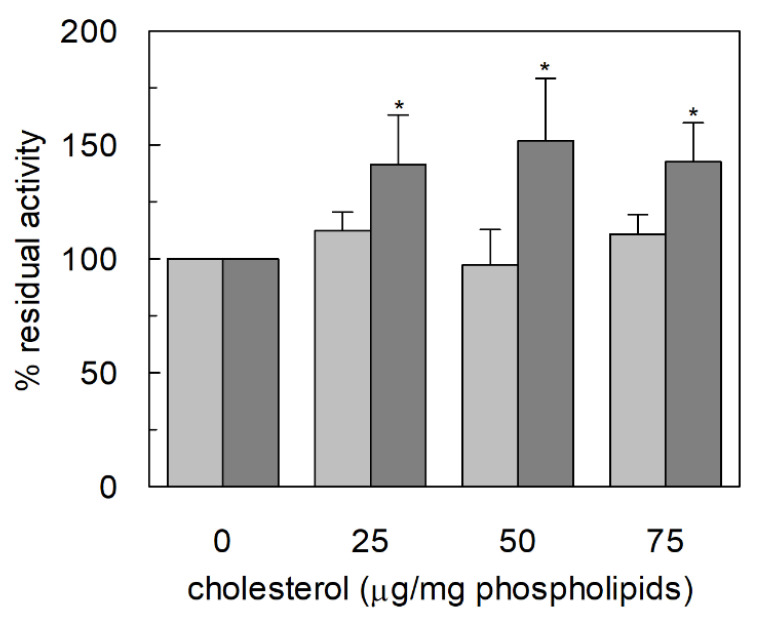
Dependence on cholesterol of the [^3^H]-glutamine unidirectional transport by ASCT2 WT and C467A mutant in proteoliposomes. Purified WT (light grey) and C467A (dark grey) proteins were reconstituted in proteoliposomes prepared with the indicated amounts of cholesterol per mg phospholipids, as described in Materials and Methods. The transport was started by adding 500 µM [^3^H]-glutamine in the presence of 50 mM Na-gluconate to proteoliposomes; the transport was stopped after 30 min according to the stop inhibitors method. The transport activity was calculated as percent of residual activity with respect to condition without cholesterol. * Significantly different from the control sample as estimated by Student’s *t*-test (*p* < 0.05).

**Figure 3:**
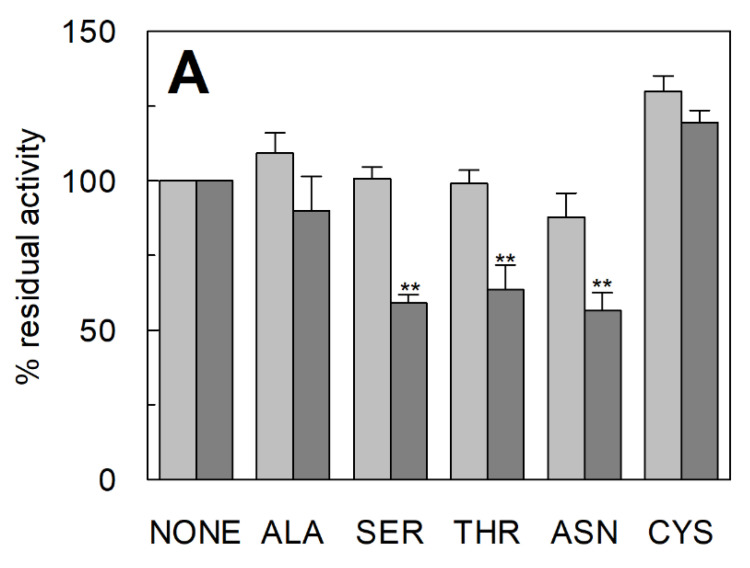

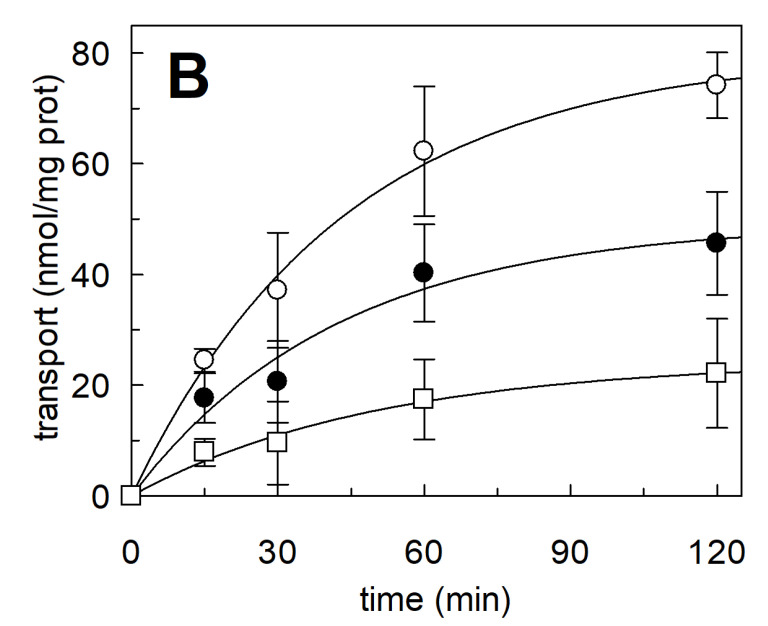
Specificity of unidirectional transport rection mediated by ASCT2 WT and C467A mutant in proteoliposomes. In (**A**), the effect of externally added amino acids on the [^3^H]-glutamine unidirectional transport by ASCT2 WT and C467A mutant is shown. Purified WT (light grey) and C467A (dark grey) proteins were reconstituted in proteoliposomes, as described in Materials and Methods. The transport was started by adding 500 µM [^3^H]-glutamine and 50 mM Na-gluconate in the presence of 10 mM indicated amino acids. The transport was stopped after 60 min according to the stop inhibitors method. The transport activity was calculated as percent of residual activity with respect to condition without cholesterol. ** Significantly different from the control sample as estimated by Student’s *t*-test (*p* < 0.01). The absolute values of the control conditions (without externally added amino acids) are: 15.6 ± 3.9 nmol/mg prot/30 min and 43.8 ± 7.15 nmol/mg prot/30 min, for WT and C467A, respectively. In (**B**), time course analysis of [^3^H]-glutamine and [^3^H]-serine unidirectional transport. The proteoliposomes were reconstituted with WT (□) or C467A mutant (‛, →), as described in Materials and Methods. The transport was started by adding 500 µM [^3^H]-glutamine (‛) or [^3^H]-serine (□, →) and 50 mM Na-gluconate to proteoliposomes. The transport was stopped at indicated times according to the stop inhibitor method. In (**A**,**B**), results are means ± SD from three independent experiments.

**Figure 4:**
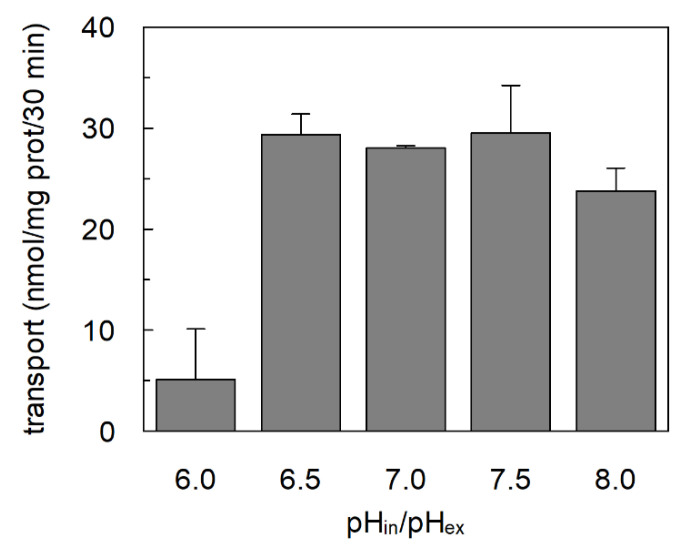
Effect of pH on [^3^H]-glutamine unidirectional transport by C467A reconstituted in proteoliposomes. The reconstitution was performed as described in Materials and Methods except that 20 mM HEPES Tris at the indicated pH was used. The transport was measured adding 500 μM [^3^H]-glutamine prepared with 20 mM HEPES Tris at the indicated pH, and 50 mM external Na-gluconate. The transport was stopped after 30 min, according to the stop inhibitor method. Results are means ± S.D. from three independent experiments.

The unidirectional transport reaction mediated by C467A was further characterized by investigating some key functional features of the ASCT2 mediated transport. At first, the dependence on cholesterol incorporated in the proteoliposome membrane was studied, previously described for the ASCT2 mediated antiport [16]. Interestingly, the glutamine unidirectional transport mediated by C467A retained the stimulation by cholesterol; on the contrary, the small unidirectional transport observed in the WT was completely insensitive to the lipid (Figure 2), similarly to the lack of Na^+^-dependence (Figure 1B). Then, the specificity of the unidirectional transport was evaluated by adding other ASCT2 substrates, as inhibitors, together with [^3^H]-glutamine during the transport assay (Figure 3A). In good agreement with the canonical ASCT2-mediated transport reaction, serine, threonine, and asparagine behaved as good inhibitors of the glutamine unidirectional transport mediated by C467A mutant, whereas alanine exerted a slight, if any, inhibition on the C467A unidirectional transport. It is worthwhile to note that, the activity of the WT protein was not affected by any of the externally added amino acids, further highlighting that the low WT-mediated unidirectional transport cannot be considered an ASCT2-mediated process. Interestingly, cysteine, which is not a substrate of ASCT2, was not able to inhibit the C467A-mediated unidirectional transport. This result confirmed that cysteine is not transported and that it has a specific inhibitory effect on the antiport reactions mediated by both WT and C467A, as previously reported [12,27]. Following the above described results, the unidirectional transport of [^3^H]-serine in C467A-reconstituted proteoliposomes was also measured (Figure 3B). The time course analysis revealed that the unidirectional transport of serine is less efficient than that of glutamine, but still greater than the WT-mediated [^3^H]-serine unidirectional transport (Figure 3B). Finally, the dependence of [^3^H]-glutamine uptake on pH was evaluated with an optimum reached between pH 6.5 and 7.5, as in the case of the antiport reaction [15] (Figure 4).

### 2.3. Kinetic Characterization of C467A Unidirectional Transport in Proteoliposomes

**Figure 5:**
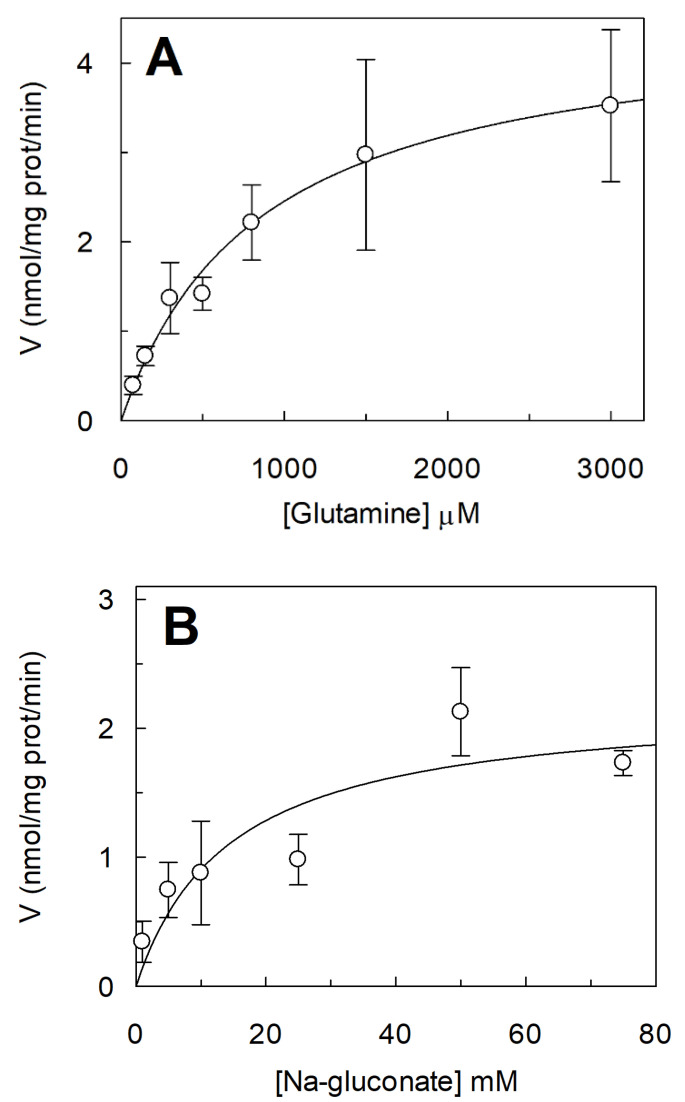
Kinetic analysis of [^3^H]-glutamine unidirectional transport by C467A reconstituted in proteoliposomes. In (**A**), dependence of the transport rate of C467A reconstituted in proteoliposomes on external [^3^H]-glutamine. The reconstitution was performed as described in Materials and Methods. The transport rate was measured by adding indicated [^3^H]-glutamine concentrations to proteoliposomes in the presence of 50 mM Na-gluconate. In (**B**), dependence of the transport rate of C467A reconstituted in proteoliposomes on external Na^+^-gluconate. The reconstitution was performed as described in Materials and Methods. The transport rate was measured by adding indicated Na-gluconate concentrations to proteoliposomes in the presence of 500 μM [^3^H]-glutamine. In (**A**,**B**), data were plotted according to Michaelis–Menten equation and results are means ± S.D. of three independent experiments.

Kinetics parameters of C467A-mediated unidirectional transport were measured (Figure 5). At first, the dependence of transport rate on increasing glutamine concentrations was evaluated and a Km of 0.85 ± 0.48 mM was obtained; this value is close to the Km measured for the C467A-mediated antiport [27]; indeed, the mutation C467A strongly affects external Km towards glutamine given the crucial role played in substrate recognition and binding [7,27]. Furthermore, the dependence of the transport rate on Na^+^ concentration was measured (Figure 5B). A Km value of 14.4 ± 7.6 mM was derived from the plotted data; this value is in the same order of magnitude of that measured for the C467A antiport reaction [15]. 

### 2.4. In Silico Analysis of hASCT2 Transport Cycle

**Figure 6:**
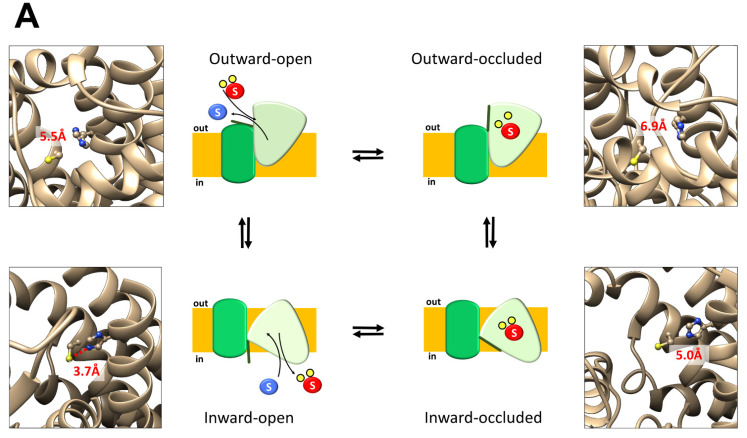

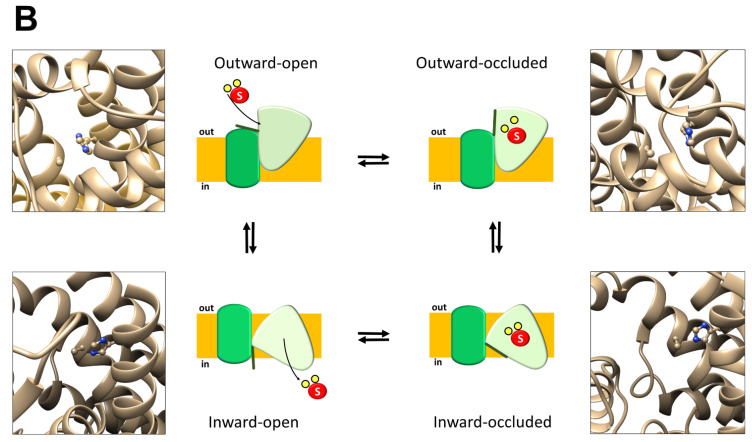
The hASCT2 predicted transport cycle. In the outward-open state, the hASCT2 binding site is accessible to the substrate to enter; after the binding of substrate, HP2 closes, and ASCT2 transit into the outward-occluded state. Subsequently, the transport domain moves towards the cytoplasm forming the inward-occluded state. After that, ASCT2 transits into the inward-open allowing substrate release into the cytoplasm. The last step is the transition towards the outward-open state that completes the cycle. The sketches of each step of the transport cycle are associated with the picture of the binding site derived from the 3D structures in the corresponding conformational state (see text). In (**A**), the antiport transport cycle of the WT protein; cysteine 467 and histidine 313 are depicted with a ball and stick representation. The distances between these two amino acids are reported in red. In (**B**), the unidirectional transport cycle of the C467A mutant protein. The C467 residue has been mutated in silico to alanine using UCSF Chimera 1.13.1 software; alanine 467 and histidine 313 are depicted with a ball and stick representation. In the figure, the sketch of one representative ASCT2 monomer with scaffold domain in dark green, scaffold domain in light green, internal substrate as blue “S”, external substrate as red “S” and 2 Na^+^ ions in yellow balls. The membrane is indicated in orange and oriented with indication of “in” and “out”. In the boxes, images of 3D structures were generated using UCSF Chimera 1.13.1 software.

The appearance of the unidirectional transport in the ASCT2 lacking the thiol group in position 467 (C467A), was further studied by analyzing the structures of ASCT2 in the various conformation of the transport cycle (Figure 6). The 3D structure of hASCT2 has been recently solved by CryoEM in two different conformations: inward occluded (PDB: 6GCT) and inward open (PDB:6RVY). The inward open structure has been obtained using a mutant of ASCT2 in which C467 was substituted with arginine. The model shown in Figure 6 has been retro-mutated from R467 to C467 for the analysis. Two other conformations, outward open and outward occluded, were inferred by homology models built on the basis of the crystal structure of bacterial Glt_Ph_ (PDB:2NWW) and of the human EAAT1 (PDB:5LLU), respectively. Then, a specific conformation of ASCT2 has been assigned to each step of the transport cycle (Figure 6), as previously performed by the group of Ryan [6]. Interestingly, the comparison among the four structures highlighted a change in the position of the key C467 residue in each of the conformations (Figure 6A); only in the inward-open conformation, the residue C467 is close enough (3.7 Å) to a second residue, namely H313, to form an H-bond [29]. In the other three conformations the H-bond could not be formed due to a higher distance. Moreover, the in silico mutagenesis confirmed that the absence of thiol group at the position 467 hampers the H-bond formation with consequent appearance of the unidirectional transport mechanism (Figure 6B). 

## 3. Discussion

The resolution of ASCT2 3D structures, achieved in recent years, highlighted that the transport occurs with a one-gate elevator mechanism in which the transport domain slides over the scaffold domain with the consequent translocation of amino acids (Figure 6) [30]. In the present work, we identify one of the molecular components responsible for the amino acid antiport coupling that was so far, a still unsolved issue in the transport mechanism. This novel finding represents an important progress in the molecular basis of the ASCT2 transport mechanism. The substrate binding site of human ASCT2 is formed by the residues S351, S353, D464, N471, and C467 [22,23]. In particular, previous studies showed that the latter residue is crucial for substrate recognition and binding and is exclusively present in ASCT2, but not in the EAAT members of the family. However, differently from other cases, the substitution of this residue with alanine is not disruptive for the protein activity, suggesting that C467 may play an additional role in the overall transport process. From the data here reported, a role of C467 in the substrate binding from the internal side emerges, which is devoted to facilitating the release of an H-bond. This bond probably slows down the reorientation of the substrate-free transporter towards the outward conformation, according to the antiport mechanism (Figure 6). In the case of C467A, the lack of SH group generates a protein able to mediate a measurable unidirectional transport reaction. In the light of this observation, C467 is a determinant of the antiport mechanism (Figure 6A). As a proof of an actual transport phenomenon, mediated by the mutant, the unidirectional transport shows the canonical characteristics of the ASCT2 vectorial reaction: (i) it is Na^+^-dependent (Figure 1 and Figure 5B); (ii) it is stimulated by cholesterol included in the proteoliposomal membrane (Figure 2); (iii) it is inhibited by other substrates of ASCT2 (Figure 3A); and (iv) it is optimal at pH 7.0 (Figure 4). The stimulation by cholesterol was previously characterized on the ASCT2 antiport reaction [16]; it is due to a positive effect of the lipid on the transport rate, but not on the substrate binding. Therefore, the observed increase in the unidirectional flux can be ascribed to a similar mechanism, further confirming that the substrate binding site layout is not affected by interaction between cholesterol molecules and ASCT2 [16]. These features allowed us to exclude that the unidirectional transport function emerging in the mutant, could be an anomalous process caused by an alteration of the protein structure. Furthermore, in line with these observations, the ratio antiport/uniport measured for C467A mutant was very close to one indicating that, at equilibrium, antiport and unidirectional transport reactions are almost identical (Figure 1C). It is worthwhile to note that, the experimental conditions specifically set up for comparing antiport and unidirectional transport reactions (see Section 2.1) were aimed at preventing the accumulation of radioactivity inside proteoliposomes downhill the concentration gradient of substrates, that is normally imposed to amplify the transport signal of the antiport reaction, and to improve the experimental analysis [15,23]. In agreement with that, under the experimental conditions described in this work, the lower transport signals caused, in some instances, higher fluctuations in the data. Finally, the kinetic parameters measured for the unidirectional transport reactions (Figure 5) are in the same order of magnitude as those previously reported for the antiport reaction [15,27]. It is important to highlight that the WT protein does not catalyze a unidirectional transport; indeed, the little flux of [^3^H]-glutamine in WT-harboring proteoliposomes, overlaps the one measured in the absence of extraliposomal Na^+^ in C467A (Figure 1B). Moreover, the WT process is also anomalous in terms of substrate specificity and response to cholesterol. These features suggest that the low [^3^H]-glutamine unidirectional flux in WT proteoliposomes could be attributable to a leakage phenomenon rather than to a conventional ASCT2-mediated transport reaction. It cannot be excluded that the glutamine leakage might be part of the channel property described for the SLC1 transporters that occurs in specific conditions and is normally measured as Cl^−^ or SCN^−^ flux [6]. An intriguing difference between unidirectional and antiport reactions in C467A mutant is the lack of inhibition by cysteine externally added to proteoliposomes (Figure 3A). This result seems in contrast to a previous study, showing that cysteine is a potent inhibitor of the antiport function of both WT and C467A proteins [27]. However, it has to be stressed that the inhibitory effect of cysteine on the antiport reactions is due to the induction of a fast efflux of internal substrates. Then, it is apparent that this phenomenon only inhibits the antiport; indeed, the inhibition is due to the loss of the internal counter substrate. Therefore, the lack of inhibition of the unidirectional transport by cysteine further agrees with a unidirectional transport-mediated process: in the absence of internal substrate, no efflux can be caused by externally added cysteine [15,27]. In summary, the results here presented assign a novel role to C467 besides that, previously proposed of substrate binding site layout. The role in the antiport coupling agrees with the unique presence of C467 residue in ASCT2, suggesting that the unidirectional transport activity in C467A mutant may be reminiscent of the unidirectional transport of amino acids catalyzed by EAATs, which do not possess the cysteine residue in the active site.

## 4. Materials and Methods

The *P. pastoris* wild type strain (X-33), the pPICZB vector, zeocin, Ni-NTA agarose resin were from Invitrogen (Life Technologies Monza, Italy); PD-10 columns were from Cytiva Europe Gmbh (Milan, Italy); L-[^3^H]-glutamine and L-[^3^H]-serine were from Perkin Elmer (Milan, Italy); C_12_E_8_ was from TCI Europe (Zwijndrecht, Belgium); cholesterol, Amberlite XAD-4, egg yolk phospholipids (3-sn-phosphatidylcholine from egg yolk), Sephadex G-75, L-glutamine, L-serine, and all the other reagents were from Merck Life Science (Milan, Italy). 

### 4.1. Recombinant Production of hASCT2 WT and C467A Mutant

The C467 residue was mutated to Ala by PCR overlap extension method, as previously described [27]. Then, to obtain the recombinant hASCT2-His6 WT and C467A mutant, 10 µg of pPICZB-ASCT2-6His WT or mutant construct was linearized with PmeI and used to transform *P. pastoris* wild type strain X-33 by electroporation [15,31]. Transformed *P. pastoris* cells were selected using YPDS agar plate containing 2000 µg/mL zeocin. For large scale protein production, transformed *P. pastoris* cells were inoculated in the BMGY medium and grown at 30 °C under rotatory stirring. *P. pastoris* cells were centrifuged and resuspended at final OD of 1 in 250 mL BMMY medium containing 0.5% methanol. The cells were placed in a 2 L conical flask and grown in the same medium at 30 °C for 3 days under rotatory stirring. Fresh methanol was added every 24 h. To isolate the *P. pastoris* membrane fraction, 30 g of cells were resuspended in 300 mL buffer containing 50 mM Tris HCl pH 7.4, 150 mM NaCl, 2 mM β-mercaptoethanol, and 0.5 mM PMSF. This cell suspension was loaded in the chamber of the bead beater (BioSpec Products, Oklahoma, USA) for disruption with glass beads (0.5 mm) for 5 min cycle, reaching 90% of cell disruption. Then, the broken cell suspension was centrifuged in a JA10 rotor at 10,000× *g* for 30 min at 4 °C to remove cell debris and unbroken cells. The supernatant, containing membrane and cytosolic fraction, was centrifuged again in a JA 30.50 rotor at 45,000× *g* for 90 min at 4 °C. The resulting membrane pellet was washed with urea buffer (5 mM Tris HCl pH 7.4, 2 mM EDTA, 2 mM EGTA and 4 M urea) and subjected to another centrifugation cycle, as described above. To reach a concentration of about 400 mg/mL, the washed membrane fraction was resuspended in a buffer composed of 25 mM Tris HCl pH 7.4, 250 mM NaCl, 2 mM β-mercaptoethanol, and 10% glycerol and homogenized using a potter homogenizer. Membrane fractions were stored at −80 °C before solubilization.

### 4.2. Solubilization and Purification of hASCT2 WT and C467A Mutant

For large-scale solubilization and purification of hASCT2 WT protein and mutant C467A, about 1.2 g of washed membranes (400 mg/mL) were solubilized using a buffer containing 25 mM Tris HCl pH 7.4, 250 mM NaCl, 6 mM β-mercaptoethanol, 10% glycerol, 1 mM L-glutamine, and 2% C_12_E_8_ (w/w). The membranes were incubated with the solubilization buffer for 3 h at 4 °C under rotatory stirring. The solubilized membranes were centrifuged at 18,000× *g* for 45 min at 4 °C and the supernatant was applied to 2 mL Ni-nitrilotriacetic acid (NTA) agarose resin pre-equilibrated with a buffer containing 20 mM Tris HCl pH 7.4, 300 mM NaCl, 10% glycerol, 6 mM β-mercaptoethanol, 0.03% C_12_E_8_, 1 mM L-glutamine, and 50 mM imidazole. The resin was incubated, with gentle agitation, at 4 °C over-night to allow specific binding of recombinant hASCT2. After incubation, the Ni-NTA resin was packed by gravity into a glass-column and washed with 30 mL of the above described equilibration buffer. Protein was eluted in a buffer containing 20 mM Tris HCl pH 7.4, 300 mM NaCl, 10% glycerol, 6 mM β-mercaptoethanol, 0.03% C_12_E_8_, 1 mM L-glutamine, and 500 mM imidazole. Fractions of 1 mL were collected, and 2.5 mL of the purified protein was desalted on a PD-10 desalting column, pre-equilibrated with a buffer composed of 20 mM Tris HCl pH 7.4, 100 mM NaCl, 10% glycerol, 6 mM β-mercaptoethanol, 0.03% C_12_E_8_, and eluted in 3.5 mL of the same buffer.

### 4.3. Inclusion of Cholesterol in Liposome Preparation

Then, 7.5 mg of cholesterol (unless differently indicated in the figure legends) were added to 100 mg of egg yolk phospholipids and solubilized with 1 mL chloroform. After incubation under rotatory stirring (30 °C 5 min 1200 rpm) solution is dried using rotavapor. The dried lipid film was resuspended in 1 mL water to a 10% final concentration. Unilamellar liposomes were prepared by two sonication cycles of 1 min (1 pulse ON and 1 pulse OFF, 40 W) with a Vibracell VCX-130 sonifier, as previously suggested [32], and used for reconstitution procedure.

### 4.4. Reconstitution of the Recombinant hASCT2 into Liposome 

The purified hASCT2 WT and C467A proteins were inserted in liposomal membrane by employing the detergent removal approach, in a batch wise procedure, as previously described [15,28]. In brief, a reconstitution mixture was prepared, composed of: 5 µg of the purified hASCT2 WT or C467A; 5 µL of 0.3 M EDTA; 120 µL 10% C_12_E_8_; 100 µL of 10% (w/v) egg yolk phospholipids (prepared without or with cholesterol, as described in Section 2.4); 100 mM sucrose to balance external osmolarity; 20 mM HEPES Tris pH 7.0 (or different pH, as specified in the figure legend); in a final volume of 700 µL. The mixture was then incubated with 0.5 g Amberlite XAD-4, for detergent removal from mixed micelles (40 min, 23 °C, 1200 rpm in thermomixer). All the operations were performed at room temperature. In the case of antiport experiments, 0.5 mM glutamine was added to the reconstitution mixture.

### 4.5. Transport Measurements

To remove the external compounds, 600 µL of proteoliposomes was passed through a Sephadex G-75 column (0.7 cm diameter × 15 cm height) pre-equilibrated with a buffer containing 20 mM HEPES Tris pH 7.0. The eluate was collected and divided in 100 µL samples used for transport assay. The uptake was started by adding 500 µM [^3^H]-glutamine (or other radiolabeled substrates as indicated in the figure legends), together with 50 mM Na-Gluconate, at room temperature. The transport reaction was stopped at the indicated times adding 1 mM HgCl_2_. In control sample, the same inhibitor was added at time zero, according to the inhibitor stop method previously described [33]. Then, 100 µL samples was passed through a Sephadex G-75 column (0.6 diameter × 8cm height), buffered with 50 mM NaCl, to separate the external from the internal radioactivity. Liposomes were eluted with 1 mL of the same buffer and collected in a 3 mL of scintillation mixture, vortexed and counted. The experimental values were analyzed by subtracting to each sample the respective blanks. The initial transport rate, expressed as nmol⋅min^−1^⋅mg protein^−1^, was measured by stopping the reaction after 20 min, i.e., within the initial linear range of radiolabeled substrate uptake. 

### 4.6. Data Analysis

Results are expressed as means ± SD. The software GraFit 5.0.13 was used to plot data in Michaelis–Menten and in first-order rate equations. Then, kinetic parameters and initial transport rate were calculated from plotted data. The statistical significance was assessed by Student’s test for *p* < 0.05 and *p* <0.001 as specified in the figure legends.

### 4.7. Other Methods 

Protein amount was measured by densitometry of SDS-PAGE stained by Coomassie Blue, using the ChemiDoc imaging system equipped with Quantity One software (Bio-Rad Laboratories). Molecular visualization of 3D structures was performed with the UCSF Chimera 1.13.1 software [34] (Resource for Biocomputing, Visualization, and Informatics, University of California, San Francisco, CA, USA). 

## Data Availability

The data presented in this study are available on request from the corresponding author.
